# Pulmonary vessel volume in idiopathic pulmonary fibrosis compared with healthy controls aged > 50 years

**DOI:** 10.1038/s41598-023-31470-6

**Published:** 2023-03-17

**Authors:** Joyce John, Alys R. Clark, Haribalan Kumar, Alain C. Vandal, Kelly S. Burrowes, Margaret L. Wilsher, David G. Milne, Brian Bartholmai, David L. Levin, Ronald Karwoski, Merryn H. Tawhai

**Affiliations:** 1 Auckland Bioengineering Institute, The University of Auckland, Private Bag 92019, Auckland, New Zealand; 2 Department of Statistics, The University of Auckland, Auckland, New Zealand; 3 Respiratory Services, Auckland City Hospital, Auckland, New Zealand; 4 Radiology, Auckland City Hospital, Auckland, New Zealand; 5 Radiology, Mayo Clinic, Rochester, MN USA

**Keywords:** Prognostic markers, Respiratory tract diseases, Image processing

## Abstract

Idiopathic pulmonary fibrosis (IPF) is characterised by progressive fibrosing interstitial pneumonia with an associated irreversible decline in lung function and quality of life. IPF prevalence increases with age, appearing most frequently in patients aged > 50 years. Pulmonary vessel-like volume (PVV) has been found to be an independent predictor of mortality in IPF and other interstitial lung diseases, however its estimation can be impacted by artefacts associated with image segmentation methods and can be confounded by adjacent fibrosis. This study compares PVV in IPF patients (N = 21) with PVV from a healthy cohort aged > 50 years (N = 59). The analysis includes a connected graph-based approach that aims to minimise artefacts contributing to calculation of PVV. We show that despite a relatively low extent of fibrosis in the IPF cohort (20% of the lung volume), PVV is 2–3 times higher than in controls. This suggests that a standardised method to calculate PVV that accounts for tree connectivity could provide a promising tool to provide early diagnostic or prognostic information in IPF patients and other interstitial lung disease.

## Introduction

Idiopathic pulmonary fibrosis (IPF) is an interstitial lung disease (ILD) that is characterised by progressive fibrosing interstitial pneumonia and an associated progressive and irreversible decline in lung function and quality of life. The time to diagnosis of IPF from onset of first symptoms is more than 12 months in about half of patients^1^, and median survival after diagnosis is 3–5 years^2^. Identifying patients who are the most at risk of rapid disease progression is critical, yet the course of the disease for individual patients remains difficult to predict. Assessment of disease severity and prognosis has been guided by clinical measurements of lung function and visual scoring of imaging. However, these are not sufficiently sensitive to predict patient-specific change in lung function or progression of disease on imaging over time. New objective and repeatable measures are therefore needed.

Objective quantitative analysis of thoracic high-resolution computed tomography (HRCT) imaging has shown promise for patient prognosis and staging. For example, recent studies using automated texture-based quantitation of lung fibrosis on HRCT have found that early structural changes on HRCT in IPF are predictive of decline in lung function^3^. Similarly, interstitial features identified using the automated tissue texture analysis software ‘CALIPER’ have been shown to have greater prognostic accuracy than visual CT scoring^4^. In particular, the extent of honeycombing and estimation of pulmonary vessel-like volume (PVV) were found to be independent predictors of mortality in IPF. Estimated PVV has also been shown to be associated with diffusion limitation for carbon monoxide (D_L_CO) in systemic sclerosis-associated ILD, and to be a strong predictor of outcome in unclassifiable ILD^5^. Several contributing mechanisms have been suggested for the larger PVV in patients who have worse prognosis^4^: (1) diversion of blood flow to areas of tissue that appear normal or mildly abnormal resulting in sustained recruitment and distension, (2) dilation of blood vessels due to increased tethering pressure on the vessels from a stiffer lung tissue, and (3) increased pleuro-parenchymal and broncho-pulmonary arterial anastomoses^6^. It has further been suggested that vascular alterations in IPF could be the first pathological lesions upon which fibrosis builds idiopathically^7–11^. Pulmonary hypertension (PH) is often a late stage feature of IPF^12^ and is well known to worsen prognosis across a range of ILDs^13^. Higher blood pressures in PH could theoretically increase PVV via vessel distension, however PVV does not appear to be associated with PH in the IPF cohorts that have so far been studied^14^.

The mean age of IPF patients is 65 years^15,16^. The healthy lung experiences changes to the tissue structure and vasculature with older age that could influence PVV^17^. Therefore, quantifying the difference between the normal healthy older lung and IPF is important for understanding the relevance of PVV to a lung disease that is largely prevalent in older people. This will also provide a first step towards identifying the specific mechanisms that are responsible for increased PVV in IPF and other ILDs. Further, it is likely that the estimation of PVV depends on the specific image processing methods and assumptions that are used in its derivation. For example, reticular abnormalities can appear as vessel-like structures^18^, and arteries or veins that are adjacent to each other could be counted as a single vessel with a large cross-sectional area on a 2D image. It is not clear whether these artefacts markedly influence the estimation of PVV or the interpretation of its association with prognosis. To address these two limitations, in the current study we compare estimated PVV between patients diagnosed with IPF and healthy controls aged 50–93 years. An over-50-years control group is selected for consistency with the average age of the IPF cohort. Estimates of PVV and its sub-volumes are compared between a method that does not explicitly exclude segmentation artefacts, and a method that assesses only connected, branching vessel-like structures. The correlation of PVV with the extent of fibrosis and measurements of percent predicted lung function is also analysed. Data from these analyses are provided in the Supplementary Information 1.

## Methods

Volumetric HRCT imaging and pulmonary function test (PFT) data were acquired retrospectively from patients diagnosed with IPF and from a study of healthy controls. 21 patients diagnosed with IPF were recruited from Auckland City Hospital, Auckland, New Zealand. Patients were recruited consecutively as they attended routine clinical appointments. The study included patients with stable coronary artery disease (6), mild or stable mixed aortic valve disease and normal cardiac function (2), and one that developed ischaemic cardiomyopathy during the course of their IPF disease.

All experimental protocols and use of data were approved by the Northern Health and Disability Ethics Committee (approval HDEC 15/STH/1/AM02). Data acquired as part of routine diagnostics and follow-up were used in the current study. Each IPF patient had baseline imaging and up to two follow-up scans, providing a total of 43 imaging datasets. PFTs were acquired at more frequent intervals than imaging, and PFTs at the closest date to imaging were used in the current analysis (with a mean time between imaging and PFTs of two months, and maximum interval of nine months). Volumetric HRCT and PFTs from 59 healthy subjects enrolled in a separate study^19^ with approval from the Northern Health and Disability Ethics Committee (approval HDEC, 13/NTA/41) were included as controls. Control subjects were aged over 50 years, had no smoking history, and normal lung function by ATS/ERS criteria^20^. IPF and control demographic and physiological data are given in Table 1. All imaging, experimental protocols, and use of data in the two cohorts were performed in accordance with relevant guidelines/procedures, and all participants provided informed consent.

**Table 1:** Demographic and physiological data for IPF and controls.

	Healthy controls (n = 59)	IPF-baseline (n = 21)	*p* value
Age (years)	71 ± 11	72 ± 9	0.7
Sex (M/F)	27/32 (n = 59)	17/4 (n = 21)	
BMI (kg/m2)	25.7 ± 2.9	27.3 ± 4.2	0.12
FVC%	117.8 ± 16.3	83.1 ± 16.0	< 0.001***
FEV1%	109.2 ± 15.7	86.5 ± 14.6	< 0.001***
TLC%	107.2 ± 13.7	68.6 ± 11.4	< 0.001***
DLCO%	89 ± 14.3	56.6 ± 10.6	< 0.001***
KCO% (DLCO/VA)	96.7 ± 13.7	87.1 ± 16.6	0.015*

Percent predicted values are given for FVC (forced vital capacity), FEV1 (forced expired volume in one second), TLC (total lung capacity), D_L_CO (diffusion capacity for carbon monoxide), and KCO (carbon monoxide transfer coefficient). Data are given as mean ± standard deviation. Independent t-test *p* values indicated by: *(significance at the 0.05 level), **(significance at the 0.01 level), ***(significance at the 0.001 level).

For all subjects, volumetric HRCT was acquired with slice thickness of 3 mm, slice spacing 2–3 mm, pixel resolution 0.418–0.824 mm, and reconstruction matrix 512 × 512. The total number of image slices ranged from 78 to 194. All imaging was acquired in the supine position at end inspiration without contrast enhancement. Images from control subjects were acquired on two scanners (Philips and GE Medical Systems). IPF patients were scanned in multiple centres using Siemens, Philips and GE Medical Systems. To maintain the similarity of grain noise and edge enhancement of the images, for both cohorts only data that used comparable reconstruction kernels were used. These were i31f, i40f, B31f, B40f, and B41f for Siemens, Standard and Soft recons for GE, and soft tissue (B) for Philips.

The automated tissue texture classification software CALIPER^21^ was used to automatically segment the lung surface, airways, and vessel-like structures, and to classify the lung tissue texture. Airway and vessel-like structures were identified as part of the automated software workflow so that they could be removed prior to the tissue texture analysis step. The vessel-like segmentation uses an enhancement filter based on the Hessian matrix to identify tubular structures, which is a widely used method^22,23^. Following removal of airway and vessel-like structures, each remaining image voxel was classified as normal, honeycomb, ground-glass, reticular, severe low attenuation area (LAA), moderate LAA, or mild LAA. The volume of tissue in regions identified as honeycomb, ground-glass, and reticular was summed and divided by the total imaged lung volume to give percent fibrosis by volume.

Pulmonary vessel-like volume (PVV) and three vessel-like sub-volumes were estimated using two methods. The first used the vessel-like structures from the primary image segmentation and summed the volume of structures based on their cross-sectional area within each image plane to give PVV5 (structures with cross-sectional area on 2D images of < 5 mm^2^), PVV10 (cross-sectional area between 5 and 10 mm^2^) and PVV10 + (cross-sectional area > 10 mm^2^). Volumes were calculated by multiplying the cross-sectional area in the image by the image slice thickness. This method has been used in prior studies to estimate PVV^4,5,14,24^. The volumes calculated using this method are referred to here as using ‘unfiltered’ data. Using cross-sectional area on a 2D image to partition the vessel-like structures essentially assumes orthogonality of the vessel direction to the image plane; and, using slice thickness as the multiplier for volume assumes that every structure in a plane is continuous through an image slice. The second estimation method aimed to minimise error from non-orthogonal vessels and non-continuous structures. 3D connected component thresholding and morphological operations were used to produce a graph-based structure that retains connected branching vessels and neglects small unconnected clusters of voxels. The centerlines of the vessels were extracted using the Python-based library ‘scikit-image’^25^. Unconnected clusters of voxels (presumed to include misclassified reticular pattern) were removed using conditional voxel removal by Euclidean distance transform^26^ and the Hann window^27^ of the lung mask. The ‘denoised’ centerline was used to generate connected graphs, i.e. connected tree-like structures. The bifurcation points between vessels were defined as nodes and the line joining two nodes was defined as a connecting edge. The radius for each vessel branch was calculated using the Euclidean distance transform matrix of the filtered vessel-like mask. Thus, the whole vessel mask was converted into a connected graph. The Python-based open source library ‘networkX’^28^ was used for this purpose. Vessel-like volume estimates using the second method are referred to as ‘graph-based’.

### Statistical analysis

#### Data pooling for IPF

In this analysis the data from baseline and follow-up imaging for the IPF group together totalled to 43 data points. Estimated PVV for the IPF cohort, unless stated otherwise, consisted of both the baseline and follow-up data points.

#### Linear mixed effect models

Linear mixed effects models (LMM) were used for statistical analysis to account for the random effect in the IPF cohort caused by acquiring data at multiple timepoints in the same patients. The dependence between baseline and follow-up imaging was accounted for using LMM-implemented variance components. LMMs account for dependence between observations measured on the same individuals and, as the case may be, during the same visit. Variance was estimated using the classical sandwich estimator^29^ to avoid assumptions of residual normality. Coefficient estimates and standard errors of the predictor variables on the response variable were used to test the statistical significance of the relationships. Results were considered statistically significant for *p* < 0.05. All analyses were performed using SAS software version 9.4 (SAS Institute, Cary, NC).

#### Comparisons

PVV, PVV5, PVV10, and PVV10 + estimates were compared between the unfiltered and graph-based methods, and between the IPF and control cohorts. Raw standard deviations were obtained from the square rooted sum of the within- and between-participant variance in the IPF cohort. LMMs were used to compare the means of the PVV and PVV sub-volume estimates across cohorts as well as between estimation methods, using the visit number nested under the participant as random intercepts. Graph-based PVV sub-volume estimates were regressed on graph-based PVV estimates in interaction with the cohort, with participants as random intercepts. Graph-based PVV estimates were regressed on fibrosis extent and measurements of lung function in interaction with the cohort, with participants as random effects.

## Results

### Patient and control data

The IPF cohort had a higher proportion of males than the control cohort, which is consistent with the higher incidence of IPF in males. Results for PVV estimates by sex are given below. Age and BMI were not different between controls and IPF (Table 1). All percent predicted values of lung function were lower on average in the IPF group (*p* < 0.05).

### The impact of filtering segmentation artefact on estimation of vessel-like volumes

**Figure 1:**
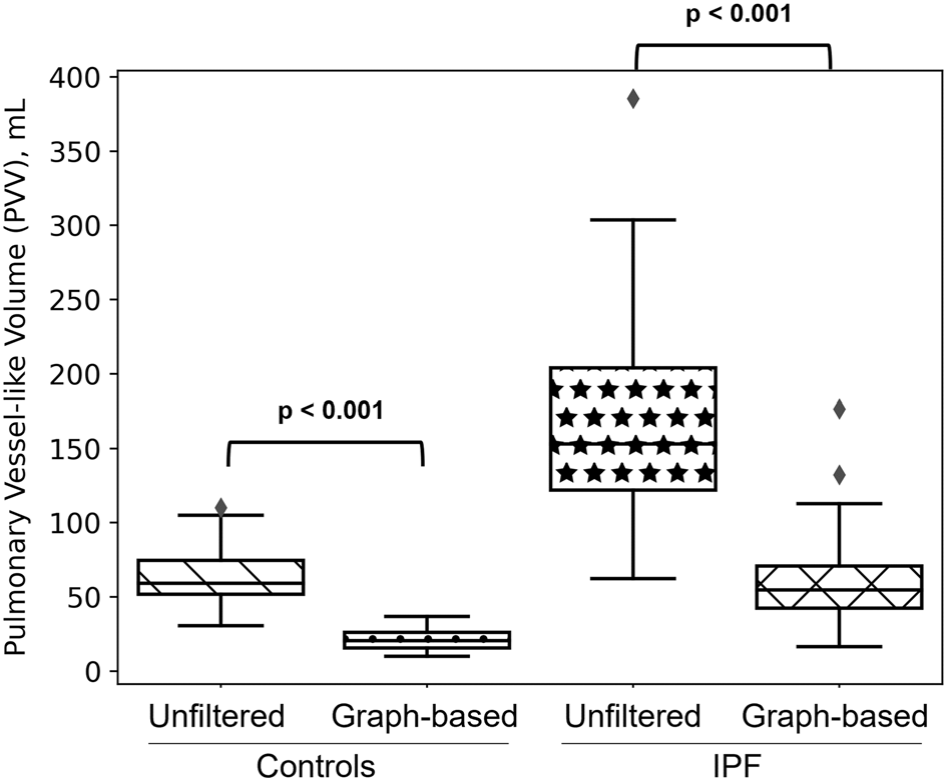
The impact of filtering segmentation artefacts on the estimation of pulmonary vessel-like volume (PVV) for IPF (baseline and follow-up data pooled) and controls. The four bar charts show PVV estimates for unfiltered and graph-based methods in controls and IPF. Graph-based PVV was lower in both IPF and controls. Statistically significant differences in volumes between the two cohorts were seen for both methods.

Figure 1 compares total PVV estimated from the unfiltered vessel-like mask and the graph-based method for IPF and controls. PVV was reduced to 37% and 34% of the unfiltered values for IPF and controls, respectively, when using the graph-based method (*p* < 0.001). The unfiltered estimated PVV values were 164.3 ± 68.6 mL and 63.9 ± 17.3 mL for IPF and controls, respectively, compared with graph-based estimates of 60.9 ± 33.4 mL and 22.0 ± 6.8 mL (when testing the differences, *p* < 0.001 and *p* < 0.001 respectively) .

**Figure 2:**
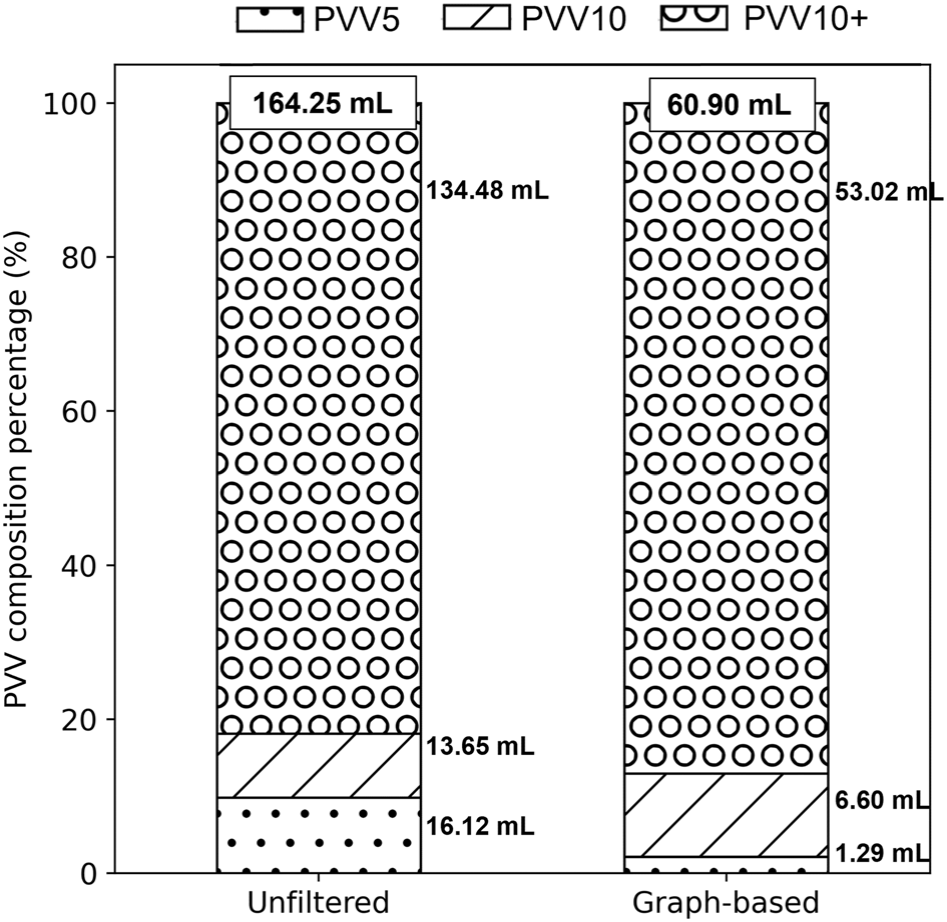
The impact of filtering segmentation artefacts on the estimation of pulmonary vessel-like (PVV) subvolumes for IPF. The bars indicate the percentage of PVV that is PVV10 + (dotted hatch), PVV10 (slashed hatch), and PVV5 (grey hatch). Numerical labels indicate the absolute values (mL) of each volume compartment. PVV5: volume of vessels with cross-sectional area less than 5 mm^2^; PVV10: volume of vessels with cross-sectional area 5–10 mm^2^; PVV10 + : volume of vessels with cross-sectional area > 10 mm^2^.

The absolute values for all PVV sub-volume estimates (Fig. 2) were smaller when using the graph-based method compared with unfiltered data in the IPF cohort (*p* < 0.001 for all). The sub-volumes as a proportion of estimated PVV were each different between the two methods: PVV10 + (unfiltered: 81.4 ± 5.0%, graph-based: 86.5 ± 6.0%; *p* < 0.001), PVV10 (unfiltered: 8.6 ± 1.8%, graph-based: 11.2 ± 4.9%; *p* < 0.001) and PVV5 (unfiltered: 10.0 ± 3.6%, graph-based: 2.4 ± 1.9%; *p* < 0.001).

The results in the following sections are for PVV estimated using the graph-based method only.

### Distributions of vessel-like sub-volumes

**Figure 3:**
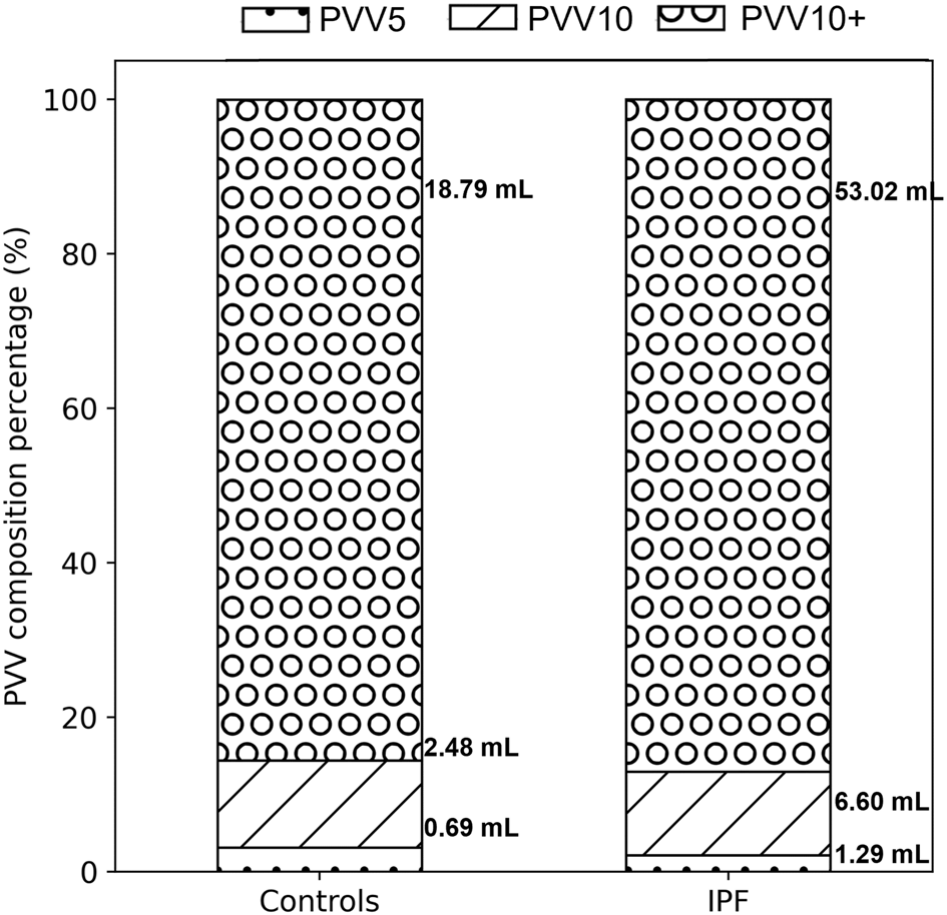
Comparison of the distribution of sub-volumes as a proportion of estimated pulmonary vessel-like volume (PVV) for IPF and controls. The stacked bars indicate the percentage of PVV that is PVV10 + (dotted hatch), PVV10 (slashed hatch), and PVV5 (grey hatch). Numerical labels indicate the absolute volumes (mL) of each volume compartment. PVV5: volume of vessels with cross-sectional area less than 5 mm^2^; PVV10: volume of vessels with cross-sectional area 5–10 mm^2^; PVV10 + : volume of vessels with cross-sectional area > 10 mm^2^.

Table 2 compares the absolute and relative (to PVV) size of each estimated sub-volume for IPF at baseline and controls. Only the baseline data for IPF were included here to investigate the difference from controls at the earliest imaging of the disease. The absolute values were higher in IPF for all sub-volumes. For the relative volumes, PVV10 + and PVV10 as a proportion of estimated PVV were not different between the cohorts, but PVV5 as a proportion of estimated PVV was lower in IPF compared with controls. Repeating this comparison using pooled baseline and follow-up data for IPF (Fig. 3) concluded the same trends and strength of relationships: *p* values were 0.002 (PVV5), < 0.001 (PVV10 and PVV10 +) for absolute values, and only PVV5 was different (*p* = 0.02) for relative values.

**Table 2:** PVV and its sub-volumes for IPF at baseline and controls, for graph-based estimates of PVV.

	Absolute volumes			Volumes relative to PVV		
	Controls (mL) (n = 59)	IPF-baseline (mL) (n = 21)	*p* value	Controls (%) (n = 59)	IPF-baseline (%) (n = 21)	*p* value
PVV	22.0 ± 6.8	52.2 ± 19.0	< 0.001***	–	–	–
PVV10 +	18.8 ± 6.1	44.8 ± 16.7	< 0.001***	85.3 ± 3.7	86.0 ± 6.6	0.63
PVV10	2.5 ± 0.9	6.2 ± 4.2	0.001**	11.4 ± 3.4	11.5 ± 5.9	0.97
PVV5	0.7 ± 0.3	1.2 ± 0.7	0.003**	3.2 ± 1.2	2.4 ± 1.2	0.01*

Sub-volumes are given as percentages of PVV. Data are given as mean ± standard deviation. Independent t-test *p* values indicated by: *(significance at the 0.05 level), **(significance at the 0.01 level), ***(significance at the 0.001 level).

Estimated PVV had positive relationships with all the subvolumes. Table 3 provides the slope estimates and respective p-values showing the significance of these relationships.

**Table 3:** Relationship between estimated PVV and its subvolumes: PVV5, PVV10 and PVV10 + .

Cohorts	PVV5		PVV10		PVV10 +	
	Slope estimate (Lower CI, Upper CI)	*p* value	Slope estimate (Lower CI, Upper CI)	*p* value	Slope estimate (Lower CI, Upper CI)	*p* value
Controls	0.020 (0.013,0.027)	< .0001***	0.090 (0.064, 0.116)	< .0001***	0.890 (0.86, 0.91)	< .0001***
IPF	0.004 (− 0.003,0.012)	0.233	0.057 (− 0.003, 0.12)	0.061	0.939 (0.87, 1.01)	< .0001***

Data are given for the slope estimate (mL/mL) and upper and lower confidence interval (CI) values (mL/mL). Independent t-test *p* values indicated by: *(significance at the 0.05 level), **(significance at the 0.01 level), ***(significance at the 0.001 level).

### Fibrosis extent and blood volumes

**Figure 4:**
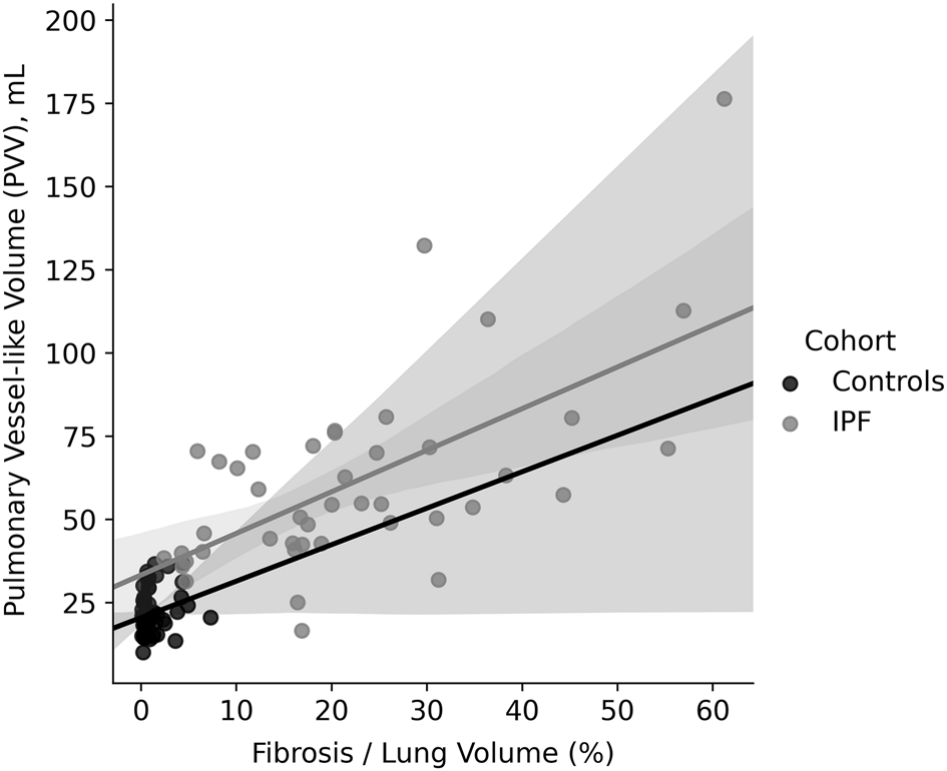
Relationships between estimated pulmonary vessel-like volume (PVV) and fibrosis as a percentage of imaged lung volume for IPF (grey) and controls (black). *P* value for IPF: *p* < 0.001 and controls: *p* = 0.04. PVV5: volume of vessels with cross-sectional area less than 5 mm^2^; PVV10: volume of vessels with cross-sectional area 5–10 mm^2^; PVV10 + : volume of vessels with cross-sectional area > 10 mm^2^.

Estimated PVV had a positive relationship with fibrosis as a proportion of total lung volume in IPF (Fig. 4, Slope estimate = 0.294 mL/%Fibrosis, *p* < 0.001). The control cohort had a very small amount of tissue automatically classified by CALIPER as ground glass (1.5 ± 2.4%), but which in this cohort is likely to be atelectasis, small areas of motion artifact, or other lung features that are within physiologic limits. A small positive relationship between estimated PVV and ‘fibrosis’ extent was found in this group (slope estimate = 0.05 mL/%Fibrosis, *p* = 0.04), and the proportion was lower than proportional lung tissue classified as fibrosis in IPF at baseline (20.5 ± 12.8%, *p* < 0.001). For the PVV sub-volumes, a relationship with fibrosis extent was only apparent for PVV10 + (Slope estimate = 0.31, *p* < 0.001) and PVV10 (Slope estimate = 1.30, *p* = 0.029): there was no relationship with PVV5.

### Vessel-like volumes and lung function

**Figure 5:**
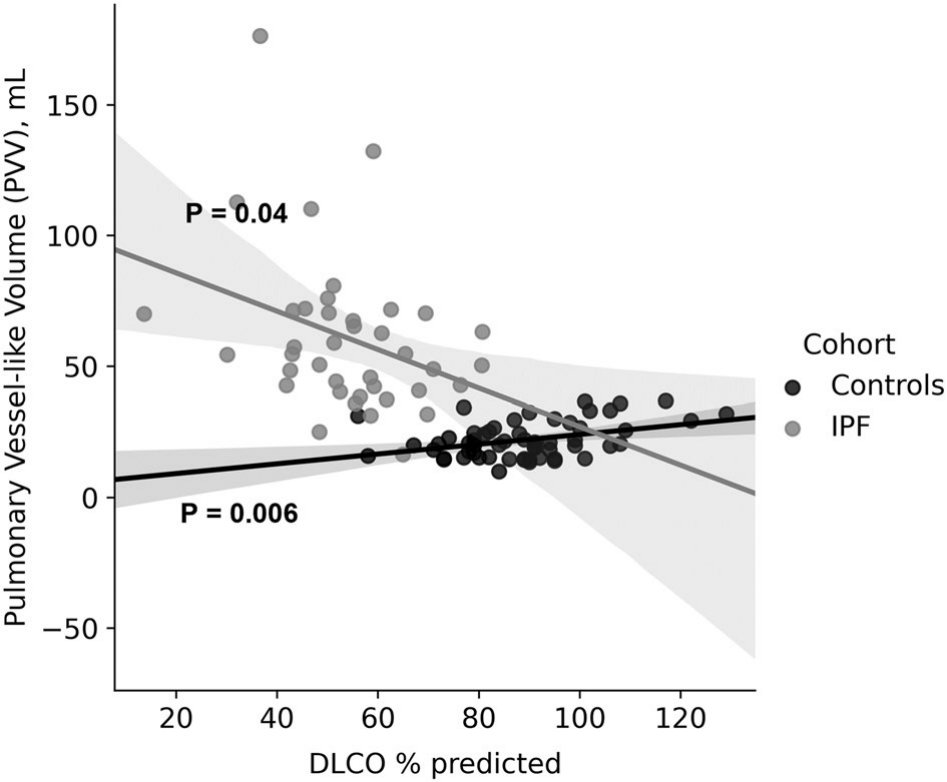
Relationships between estimated pulmonary vessel-like volume (PVV) and percent predicted diffusion capacity for carbon monoxide (D_L_CO%) for IPF (grey) and controls (black). Control PVV has a positive relationship, and IPF a negative relationship, with D_L_CO%.

The relationship between estimated PVV and gas exchange (D_L_CO% predicted, D_L_CO%) measurements of lung function is shown in Fig. 5. The strength of relationships with static (TLC% predicted), dynamic (FVC% predicted) and gas exchange (D_L_CO% predicted) measurements were tested. For both controls and IPF, a relationship was found only for PVV with D_L_CO% (Table 4). The slope of the relationship with D_L_CO% was negative in IPF and positive in controls, indicating that the larger PVV in IPF was related to declining diffusion capacity of the lung, whereas, in controls, higher PVV was related to improved gas exchange.

**Table 4:** Relationship between estimated PVV and PFT measures in control and IPF cohorts.

PFT	Cohort	Slope estimate (Lower CI, Upper CI)	*p* value
FEV1/FVC	IPF	− 0.005 (− 0.04, 0.03)	0.780
	Controls	0.038 (− 0.22, 0.30)	0.771
FVC%	IPF	− 0.068 (− 0.19, 0.05)	0.244
	Controls	0.539 (− 0.16, 1.24)	0.130
FEV1%	IPF	− 0.051 (− 0.15,0.05)	0.318
	Controls	0.631 (− 0.012, 1.27)	0.054
TLC%	IPF	− 0.075 (− 0.23, 0.08)	0.316
	Controls	0.142 (− 0.42, 0.70)	0.614
D_L_CO%	IPF	− 0.139 (− 0.27, − 0.004)	0.0437*
	Controls	0.798 (0.24, 1.35)	0.0057***
KCO%	IPF	− 0.136 (− 0.35, 0.08)	0.198
	Controls	0.198 (− 0.31, 0.71)	0.440

Data are given for the slope estimate (mL/%PFT measure) and upper and lower confidence interval (CI) values (mL/%PFT measure). Independent t-test *p* values indicated by: *(significance at the 0.05 level), **(significance at the 0.01 level), ***(significance at the 0.001 level).

*FEV1* forced expired volume at 1 s; *FVC* forced vital capacity; *TLC* total lung capacity; *D*_*L*_*CO* diffusion capacity for carbon monoxide; *KCO* transfer coefficient for carbon monoxide.

### Sex differences

Estimated PVV was smaller in females than males in the control group (F = 18.2 ± 4.3 mL, M = 26.4 ± 6.6 mL, *p* < 0.001) whereas there was no difference in IPF (F = 46.3 ± 30.6 mL, M = 53.6 ± 16.3 mL, *p* = 0.67). PVV as a percentage of lung volume (PVV/LV) was also smaller in the control females than males (F = 0.35 ± 0.12%, M = 0.49 ± 0.15%, *p* < 0.001) but not different in IPF (F = 1.36 ± 0.84%, M = 1.22 ± 0.47%, *p* = 0.63). PVV and PVV/LV were larger in IPF males than controls (both *p* < 0.001). For females, the mean values of PVV and PVV/LVV were larger in IPF, however the differences were not significant (*p* = 0.16 and *p* = 0.095 for PVV and PVV/LV, respectively).

## Discussion

The pulmonary circulation remodels in response to diseases of the lung tissue, as well as to diseases of the vasculature and airways^30–32^. Recent studies have therefore investigated the association between pulmonary vessel-like volume (PVV) on HRCT and patient outcome for a range of ILDs^4,5^, finding that PVV has potential prognostic value. Translation to use as a clinical biomarker requires understanding how PVV might differ between IPF and healthy older people. Our study compared estimates of PVV and its sub-volumes between IPF and control data from a healthy cohort of the same age range. We found that estimated PVV was more than twice as large in our IPF cohort than in controls, and was most strongly associated with extent of fibrosis but also associated with D_L_CO%, FVC%, and TLC%. Removal of very small unconnected vessel artefacts reduced PVV similarly in both cohorts, but did not reduce the strength of relationships with physiological variables or extent of fibrosis.

IPF has age-related prevalence, appearing most frequently in patients aged > 50 years^15,16^. Healthy aging is itself associated with a number of changes to the respiratory system, some of which potentially affect the pulmonary vascular volume. These include increased vascular stiffness and decreased capillary blood volume, both of which increase resistance and can cause increased blood pressure. Studies have shown association between PVV and fibrosis in IPF and other ILDs, but have not compared with healthy controls. It was therefore important to assess whether PVV quantified from HRCT is associated with age, and whether PVV in IPF is different from controls with a similar age profile. The physiology of the IPF cohort and controls were statistically distinct, with lower %predicted values of FVC, FEV_1_, TLC, D_L_CO (all with *p* < 0.001), and KCO (*p* = 0.015) in the IPF cohort, consistent with the impact of the disease on lung function.

Estimated PVV was not associated with age or %predicted lung volumes in the control group, whereas it had a positive relationship with D_L_CO% (Fig. 5). That is, a larger PVV estimate was associated with improved gas exchange in controls and abnormal gas exchange in IPF. PVV in healthy controls is likely to be proportional to the total recruited pulmonary circulatory volume and therefore to the functional gas exchange surface area, which translates to a relationship with D_L_CO%.

It is not clear whether the correlations between PVV estimate and D_L_CO% represent mechanistic relationships. Reduced D_L_CO in IPF is likely a consequence of a combination of ventilation-perfusion mismatch and reduced gas exchange surface area. A reduced surface area translates to an increased capillary contribution to pulmonary vascular resistance and therefore increased pulmonary artery pressure (PAP) which could cause distension of the larger vessels. Hypoxic pulmonary vasoconstriction in poorly ventilated regions could be a further factor in increasing pulmonary vascular resistance (PVR). A recent analysis of the prevalence of pulmonary hypertension (PH) in IPF suggests that more than 70% of IPF patients have mean PAP (mPAP) greater than 20 mmHg, but only 10% have severe elevation of mPAP to greater than 35 mmHg^33^. It is therefore unlikely that elevation in PAP by itself explains the large difference in PVV between controls and IPF that we have observed. Conducting airway volume (anatomical deadspace, VD) also increases in IPF, but not to the same extent as PVV. The more compliant vessel wall is likely to be a factor in this difference. Plantier et al.^34^ found an approximately 50% increase in VD normalised by predicted TLC in an IPF cohort compared with controls (34.2 ± 11.0 mL/L in controls, 45.3 ± 12.8 mL/L in IPF), but this was independent of D_L_CO or disease severity. Interaction between the tissue and vessels or airways is therefore important for increasing PVV and VD, but it appears that the airways and vessels have some different mechanisms driving their volume increase. The extent of fibrosis in our cohort at baseline was approximately 20% of the lung volume. Elevations in PVV estimates were not restricted to areas of the lung where fibrosis was present (predominantly basally), but was larger than in normal controls across the whole lung (data not shown). A large proportion of vessels with higher than normal volume are therefore surrounded by tissue that is ‘radiologically normal’.

Accurate segmentation of pulmonary vessels is challenging, particularly in IPF. Previous studies that have shown association between PVV and patient outcome have therefore used a pragmatic approach, estimating PVV from a vessel segmentation that is performed to facilitate the removal of vessel-like structures from the images prior to tissue texture classification. Pulmonary vessel-like volumes were then calculated by summing the product of slice thickness and 2D cross-sectional area of vessel-like structures on each imaging slice. This summation method potentially introduces error for vessels that are not close to orthogonal to the imaging plane. That is, the cross-sectional area will be overestimated in proportion to the difference from orthogonal; this can give rise to mis-classification by PVV sub-volume. Additionally, it is possible for reticular pattern to be misclassified as vessel-like when using automated methods because reticular tissue has a similar CT intensity to blood^4^. Our graph-based method was used to determine whether removing small unconnected imaging features and estimating vessel volume using the radius orthogonal to the vessel centreline affects the interpretation of PVV in IPF. We found that estimated PVV was considerably smaller when only the connected tree was retained compared with ‘unfiltered’ data, but the proportional reduction in IPF and controls—and across the sub-volumes—was generally similar (Table 2). This suggests that the elevated PVV observed in IPF is distributed approximately proportionately across the larger (i.e. > 5 mm^2^ cross-sectional area) vessels. The graph-based PVV5/PVV was smaller than the unfiltered PVV, which is reasonable considering that PVV5 is the volume most likely to be impacted by filtering small imaging features. This is also the volume that is most likely to include reticular artefacts. Jacob et al.^4^ repeated their PVV analysis with PVV5 removed, finding that PVV > 5 had strong prognostic value. Our results are consistent with this, finding significant associations between PVV and physiological measurements when filtering artefact from all of the sub-volumes. It is important to note that our filtering is a relatively aggressive approach that removes all small unconnected features. Some of these are likely to be ‘valid’ vessels for the estimation of PVV, partly explaining the much smaller volumes from our filtered compared with raw data.

Previous studies have found that the extent of fibrosis on CT is an independent predictor of mortality in IPF^35^ and indicates prognosis^36,37^. We found a positive relationship between PVV and the percentage of the lung tissue classified as fibrosis (Fig. 4). This relationship was dominated by the larger (PVV10 +) vessels; the smaller vessel sub-volumes were not correlated with fibrosis. This suggests that the relationship between fibrosis and PVV is likely to be due to the changes in the lung parenchyma in IPF resulting in vascular remodelling or distension in larger vessels, rather than a misclassification of fibrosis as vascular bed. This supports the relevance of PVV as used in previous studies.

This was a single centre study that included a relatively small number of patients: the number of patients in the IPF cohort was less than half that of controls, therefore the imaging data from baseline and follow-up scans were pooled for some of the analyses. The difference in cohort size means that while age was not statistically different between controls and IPF, the cohorts were not strictly age-matched. Baseline imaging (i.e. the first CT imaging acquired during clinical assessment) was included for all patients. This possibly weights the IPF cohort towards earlier stage disease, because follow-up imaging was not available for all patients. A much larger sample size with follow up imaging would be required to evaluate the relationship between PVV and time from baseline imaging.

Since IPF is progressive and irreversible, the baseline and follow-up scans are not identical; however they are not independent data. Pooling all the IPF data together needed to consider the case of variance structure and normality of errors. LMM was used for this reason to consider the random effect in the data caused by the multiple timepoints. The visits of each patient were not equally spaced in time (duration between timepoints for each patient were varying). Therefore, patient ID was selected as a random effect to gain strength from the repeated measure within the IPF cohort. Additionally, empirical estimators within SAS were used to avoid the assumptions on normality of errors and the variance structure. The estimates were still asymptotically unbiased under the correct model.

A further potential limitation is that the control group had close to equal numbers of male and female whereas the IPF group had only 19% females. To understand whether this could impact on our results we calculated mean PVV and the % ratio of PVV to segmented lung volume separately for males and females in each cohort. Absolute and relative values for males in the control group were larger than females, and IPF males were larger than control males; however there were no sex differences for IPF nor between females in the two groups. The low number of females with IPF and their relatively large standard deviations are a likely factor in not reaching statistical significance. Therefore, increasing their number would potentially reveal cohort differences for females and similar sex differences in IPF to those found for controls, but would not alter the between-cohort differences found in this study. That is, the significantly larger PVV in IPF males compared with control males suggests that sex-aggregated cohort differences are not likely to be because of sex differences. Sex differences in symptoms, disease burden, and outcomes have been reported for a number of lung diseases, including IPF^38^. Han et al.^39^ found that females are more likely to report dyspnea at an earlier stage of disease (as defined by %D_L_CO), and have better physical health-related quality of life (HRQL) but worse emotional HRQL than males with IPF. It is not known whether there is a physiological basis to this. If the lack of difference in PVV and PVV/LV in IPF is still present when more females are included in the analysis, this might suggest a greater impact of IPF on PVV in females than males, when compared with the lower PVV/LV in female controls.

The graph-based method discards vessel-like structures if they were not connected to the more proximal tree, which results in a lower volume of vessel-like structures being detected than when connectivity is not considered. Methods to segment the pulmonary vessels are becoming more sophisticated, for example utilising deep learning methods to automatically separate the pulmonary arteries and veins^40,41^. These advanced methods can provide more extensive segmentations than were used in the current study, as well as division of PVV into arterial and venous components that might provide more subtle information to connect local tissue remodelling with vascular changes. This would potentially provide a means to further standardise calculation of PVV, with a graph-based method providing connectivity and branching data that would allow calculation of PVV to a standardised anatomical level in the vascular tree, which may overcome limitations of vascular size and dilation leading to increased detection of blood vessels within the resolution of HRCT.

In conclusion, we have shown that the pulmonary vessel-like volume in IPF is different from healthy controls with a similar age range, and this difference remains distinct even when potential artefacts and unjoined sections of the vascular tree are removed from the data using a graph-based method. The extent of fibrosis in our IPF cohort was only 20% of the lung volume, whereas PVV was 2–3 times higher than in controls.

## Supplementary Information

Supplementary Information.

## Data Availability

All data generated and analysed in this study are provided as a table in the supplementary material.

## References

[1] Snyder LD (2020). Time to diagnosis of idiopathic pulmonary fibrosis in the IPF-PRO Registry. BMJ Open Respir. Res..

[2] Olson AL (2007). Mortality from pulmonary fibrosis increased in the United States from 1992 to 2003. Am. J. Respir. Crit. Care Med..

[3] Kim GHJ (2020). Prediction of idiopathic pulmonary fibrosis progression using early quantitative changes on CT imaging for a short term of clinical 18–24-month follow-ups. Eur. Radiol..

[4] Jacob, J. *et al.* Mortality prediction in idiopathic pulmonary fibrosis: evaluation of computer-based CT analysis with conventional severity measures. *Eur. Respir. J.* **49** (2017). 10.1183/13993003.01011-2016 27811068

[5] Jacob J (2017). Unclassifiable-interstitial lung disease: Outcome prediction using CT and functional indices. Respir. Med..

[6] Turner-Warwick M (1963). Precapillary systemic-pulmonary anastomoses. Thorax.

[7] Puxeddu, E., Cavalli, F., Pezzuto, G., Teodori, E. & Rogliani, P. Impact of pulmonary vascular volume on mortality in IPF: is it time to reconsider the role of vasculature in disease pathogenesis and progression? *Eur. Respir. J.* **49** (2017). 10.1183/13993003.02345-2016 28232417

[8] Ebina M (2004). Heterogeneous increase in CD34-positive alveolar capillaries in idiopathic pulmonary fibrosis. Am. J. Respir. Crit. Care Med..

[9] Colombat M (2007). Pulmonary vascular lesions in end-stage idiopathic pulmonary fibrosis: Histopathologic study on lung explant specimens and correlations with pulmonary hemodynamics. Hum. Pathol..

[10] Kim KH (2010). Iron deposition and increased alveolar septal capillary density in nonfibrotic lung tissue are associated with pulmonary hypertension in idiopathic pulmonary fibrosis. Respir. Res..

[11] Sangiuolo F (2015). HFE gene variants and iron-induced oxygen radical generation in idiopathic pulmonary fibrosis. Eur. Respir. J..

[12] Gross TJ, Hunninghake GW (2001). Idiopathic pulmonary fibrosis. N. Engl. J. Med..

[13] Smith JS, Gorbett D, Mueller J, Perez R, Daniels CJ (2013). Pulmonary hypertension and idiopathic pulmonary fibrosis: A dastardly duo. Am. J. Med. Sci..

[14] Jacob J (2016). Automated quantitative computed tomography versus visual computed tomography scoring in idiopathic pulmonary fibrosis: Validation against pulmonary function. J. Thorac. Imaging.

[15] Raghu G, Weycker D, Edelsberg J, Bradford WZ, Oster G (2006). Incidence and prevalence of idiopathic pulmonary fibrosis. Am. J. Respir. Crit. Care Med..

[16] Raghu G (2018). Diagnosis of idiopathic pulmonary fibrosis. An official ATS/ERS/JRS/ALAT clinical practice guideline. Am. J. Respir. Crit. Care Med..

[17] Synn, A. J. *et al.* Radiographic pulmonary vessel volume, lung function and airways disease in the Framingham Heart Study. *Eur. Respir. J.* **54** (2019). 10.1183/13993003.00408-2019 PMC7173739 31248956

[18] Chen A, Karwoski RA, Gierada DS, Bartholmai BJ, Koo CW (2020). Quantitative CT analysis of diffuse lung disease. Radiographics.

[19] Osanlouy M (2020). Lung and fissure shape is associated with age in healthy never-smoking adults aged 20–90 years. Sci. Rep..

[20] Best AC (2008). Idiopathic pulmonary fibrosis: Physiologic tests, quantitative CT indexes, and CT visual scores as predictors of mortality. Radiology.

[21] Bartholmai BJ (2013). Quantitative computed tomography imaging of interstitial lung diseases. J. Thorac. Imaging.

[22] Foruzan AH, Zoroofi RA, Sato Y, Hori M (2012). A Hessian-based filter for vascular segmentation of noisy hepatic CT scans. Int. J. Comput. Assist. Radiol. Surg..

[23] Annunziata R, Garzelli A, Ballerini L, Mecocci A, Trucco E (2016). Leveraging multiscale hessian-based enhancement with a novel exudate inpainting technique for retinal vessel segmentation. IEEE J. Biomed. Health Inform..

[24] Jacob J (2018). Predicting outcomes in idiopathic pulmonary fibrosis using automated computed tomographic analysis. Am. J. Respir. Crit. Care Med..

[25] van der Walt S (2014). scikit-image: Image processing in python. PeerJ.

[26] Jain AK (1989). Fundamentals of Digital Image Processing.

[27] Pielawski N, Wahlby C (2020). Introducing Hann windows for reducing edge-effects in patch-based image segmentation. PLoS ONE.

[28] Aric A., Hagberg, D. A. S., Swart P. J. in *Proceedings of the 7th Python in Science Conference (SciPy2008)* (ed Travis Vaught Gäel Varoquaux, Jarrod Millman) 11–15 (Pasadena, CA USA, 2008).

[29] White H (1982). Maximum likelihood estimation of misspecified models. Econometrica.

[30] Tuder RM (2017). Pulmonary vascular remodeling in pulmonary hypertension. Cell Tissue Res..

[31] Siafakas NM, Antoniou KM, Tzortzaki EG (2007). Role of angiogenesis and vascular remodeling in chronic obstructive pulmonary disease. Int. J. Chron. Obstruct. Pulmon. Dis..

[32] Harkness LM, Kanabar V, Sharma HS, Westergren-Thorsson G, Larsson-Callerfelt AK (2014). Pulmonary vascular changes in asthma and COPD. Pulm. Pharmacol. Ther..

[33] Nathan SD (2021). Impact of the new definition for pulmonary hypertension in patients with lung disease: An analysis of the United Network for Organ Sharing database. Pulm. Circ..

[34] Plantier L (2016). Increased volume of conducting airways in idiopathic pulmonary fibrosis is independent of disease severity: A volumetric capnography study. J. Breath Res..

[35] Lynch DA (2005). High-resolution computed tomography in idiopathic pulmonary fibrosis: diagnosis and prognosis. Am. J. Respir. Crit. Care Med..

[36] Edey AJ (2011). Fibrotic idiopathic interstitial pneumonias: HRCT findings that predict mortality. Eur. Radiol..

[37] Shin KM (2008). Prognostic determinants among clinical, thin-section CT, and histopathologic findings for fibrotic idiopathic interstitial pneumonias: tertiary hospital study. Radiology.

[38] Han MK (2018). Female sex and gender in lung/sleep health and disease. increased understanding of basic biological, pathophysiological, and behavioral mechanisms leading to better health for female patients with lung disease. Am. J. Respir. Crit. Care Med..

[39] Han MK (2010). Gender influences health-related quality of life in IPF. Respir. Med..

[40] Rahaghi F (2018). CT based arterial and venous morphologic changes in pulmonary hypertension associated with COPD. Eur. Resp. J..

[41] Estepar, R. S. *et al.* in *2012 9th IEEE Int Symp Biomed Imaging (ISBI).* 1479–1482 (IEEE). 10.1109/ISBI.2012.6235851 PMC3670102 23743962

